# Prognostic Value of Pretreatment Systemic Immune-Inflammation Index in Glioblastoma Multiforme Patients Undergoing Postneurosurgical Radiotherapy Plus Concurrent and Adjuvant Temozolomide

**DOI:** 10.1155/2020/4392189

**Published:** 2020-05-23

**Authors:** Erkan Topkan, Ali Ayberk Besen, Yurday Ozdemir, Ahmet Kucuk, Huseyin Mertsoylu, Berrin Pehlivan, Ugur Selek

**Affiliations:** ^1^Başkent University Medical Faculty, Department of Radiation Oncology, Adana, Turkey; ^2^Başkent University Medical Faculty, Department of Medical Oncology, Adana, Turkey; ^3^Mersin City Hospital, Radiation Oncology Clinics, Mersin, Turkey; ^4^Department of Radiation Oncology, Bahçeşehir University, Istanbul, Turkey; ^5^Department of Radiation Oncology, Koc University, School of Medicine, Istanbul, Turkey; ^6^Department of Radiation Oncology, The University of Texas, MD Anderson Cancer Center, Houston, TX, USA

## Abstract

**Objectives:**

To evaluate the potential prognostic utility of pretreatment systemic immune-inflammation index (SII) in newly diagnosed glioblastoma multiforme (GBM) patients who underwent postneurosurgical radiotherapy and concurrent plus adjuvant temozolomide.

**Methods:**

The retrospective data of GBM patients who underwent postneurosurgical radiotherapy and concurrent plus adjuvant temozolomide were analyzed. For each patient, SII was calculated using the platelet, neutrophil, and lymphocyte measures obtained on the first day of treatment: SII = platelets × neutrophils/lymphocytes. The receiver operating characteristic (ROC) curve analysis was utilized for the evaluation of optimal cut-off values for SII those linked with the outcomes. Primary and secondary endpoints constituted the overall (OS) and progression-free survival (PFS) per conveyance SII group.

**Results:**

A total of 167 patients were included. The ROC curve analysis identified the optimum SII cut-off at a rounded 565 value that significantly interacted with the PFS and OS and stratified patients into two groups: low-SII (SII < 565; *n* = 71) and high-SII (SII ≥ 565; *n* = 96), respectively. Comparative survival analyses exhibited that the high-SII cohort had significantly shorter median PFS (6.0 versus 16.6 months; *P* < 0.001) and OS (11.1 versus 22.9 months; *P* < 0.001) than the low-SII cohort. The relationship between the high-SII and poorer PFS (*P* < 0.001) and OS (*P* < 0.001) further retained its independent significance in multivariate analysis, as well.

**Conclusions:**

The outcomes displayed here qualified the pretreatment SII as a novel independent prognostic index for predicting survival outcomes of newly diagnosed GBM patients undergoing postneurosurgical radiotherapy and concurrent plus adjuvant temozolomide.

## 1. Introduction

Maximal safe resection supplanted by radiotherapy (RT) plus concurrent and adjuvant temozolomide (TMZ) (full Stupp protocol) with/without alternating electric field therapy remains to be the current gold-standard first-line treatment of medically fit glioblastoma multiforme (GBM) patients [1, 2]. Contradicting with the striking innovations in diagnostic and intraoperative neuroimaging techniques and accessible treatment modalities, the prognosis of GBM remains bleak with 5-year survival estimates of 13% even with the addition of alternating electric field therapy to adjuvant TMZ and only <5% with standard protocol, respectively [1–3].

The 2007 and more recently revised 2016 World Health Organization (WHO) grading frameworks typically assign GBM among the most threatening grade IV gliomas, principally by depending on the histological tumor phenotype, signature molecular genetic alterations, and to some degree the treatment course and results [4, 5]. Coupled with the well-known prognosticators, like the patients' age, neurologic function status, Karnofsky performance status (KPS), and Radiation Therapy Oncology Group recursive partitioning analysis (RTOG-RPA) group, this comprehensive framework provides relevant predictive and prognostic information about the prognosis of GBM patients. Nevertheless, the broad outcome variations among the patients with comparable clinicopathologic and molecular genetic properties [6], even when treated exactly with the same treatment protocols, strongly underscore the pressing demand for the search of novel biomarkers which may serve useful in the befitted forecasts of such patients' outcomes and guidance of personalized treatments. In this respect, immunity and inflammation have attained growing attention in the last two decades for their gliomagenic and malignancy potentiating properties. Hence, various immune/inflammatory blood products have been investigated either individually or in various blends for the creation of novel predictive/prognostic models in many malignancies including the GBM. For this purpose, albumin, C-reactive protein, neutrophils, lymphocytes, platelets, monocytes, neutrophil to lymphocyte ratio (NLR), platelet to lymphocyte ratio (PLR), prognostic nutritional index (PNI), and the Glasgow prognostic score (GPS) have been studied in the setting of GBM [7–11]. Another recently emerged novel score that has been tested at many tumor sites, such as the small-cell lung-and non-small-cell lung, hepatocellular, esophageal, biliary system, colorectal and urinary cancers, and acral melanomas for its prognostic value, is the systemic immune-inflammation index (SII): calculated by using the absolute measures of platelets, neutrophils, and lymphocytes obtained from the routine complete blood counts [12]. Besides being confirmed as an efficient prognostic tool in clinical studies and meta-analyses [13–16], outcomes of a recent esophageal squamous-cell carcinoma study further suggested that SII was superior over both NLR and PLR in prognosis prediction [17].

Formerly, SII has been investigated only in two studies for its preoperative discriminative performance between the low- (LGG) and high-grade gliomas (HGG), and SII levels were found to be straightly agreed with the pathologic glioma grades in both studies [18, 19]. Although neither studies addressed the prognostic worth of the SII exclusively in GBM patients, the investigators of both studies reported that the levels of SII were firmly associated with the glioma grades, namely the SII levels were significantly higher in the cohort presenting with an HGG than their LGG matches [18, 19]. Notified with these results, it is prudent to assume that the aggressive HGG histology was associated with a stronger systemic immune-inflammatory response as opposed to the more indolent LGG histology.

Notwithstanding the accessibility compelling basic evidence at numerous tumor sites, to date, surprisingly, the prognostic utility of SII has never been scrutinized in GBM patients. Hence, the current retrospective analysis endeavored to uncover the prognostic significance of SII in newly diagnosed GBM patients who underwent the postneurosurgical full Stupp protocol.

## 2. Patients and Methods

### 2.1. Eligibility Criteria

We retrospectively analyzed the clinical records of all newly diagnosed GBM patients, who underwent postneurosurgical partial brain RT with concurrent TMZ and up to 6-12 cycles of adjuvant TMZ between February 2007 and December 2017 at our institution. To be eligible, patients required to meet the following criteria: (1) pathologic proof of GBM, (2) aged 18 to 80 years, (3) Karnofsky performance score (KPS) ≥ 70, (4) no prior cranial RT and/or chemotherapy, (5) available pre- and postoperative gadolinium-enhanced magnetic resonance imaging (Gd-MRI) scans, (6) available pretreatment complete blood count tests, (7) available blood chemistry tests with adequate hematologic, renal, and hepatic functions, (8) no proof for active infection, (9) no history of chronic diseases demanding active immunosuppressive therapies, and (10) no history of second solid/hematologic cancers.

### 2.2. Ethics, Consent, and Permissions

The present study was conducted according to the principles of the by following the Helsinki Declaration and Rules of Good Clinical Practice, and the study design was approved by the Institutional Ethical Committee review board of Başkent University Medical Faculty before the acquisition of any patient information. According to our institutional standards, all patients provided written informed consent before the initiation of treatment either themselves or legally authorized representatives for the collection and analysis of blood samples, pathologic specimens, and publication of their outcomes.

### 2.3. Treatment Characteristics

As indicated by our institutional standards for GBMs, all patients were first evaluated for maximal safe neurosurgical resection and underwent this procedure if elected expedient. A postoperative 3-dimensional conformal or simultaneous integrated boost intensity-modulated RT to a total dose of 60-70 Gy (2.0 or 2.33 Gy/fx) over 6 weeks was delivered. All patients received TMZ (75 mg/m^2^/day, 7 days/week) and prophylactic trimethoprim-sulfamethoxazole against *Pneumocystis jirovecii* from the first till the last day of RT during the concurrent chemoradiotherapy phase, while the adjuvant chemotherapy comprised up to 12 cycles of maintenance TMZ (150/200 mg/m^2^/day, for 5 days, every 28 days).

### 2.4. Systemic Immune-Inflammation Index Assessment

For each patient, the SII was calculated according to Hu's original formula: SII = platelets × neutrophils/lymphocytes, by using the platelet, neutrophil, and lymphocyte measures obtained from the routine complete blood count analysis performed on the first day of concurrent RT and TMZ [12].

### 2.5. Response Assessment

Following the completion of RT and simultaneous TMZ, treatment response was assessed by utilizing Gd-MRI of the brain at every 2 months for the first and every 3 months intervals for the second follow-up years. Thereafter, Gd-MRI scans were evaluated every 6 months for the rest of the subsequent period, or more frequently if suspected clinically.

### 2.6. Statistical Analysis

The primary and secondary endpoints of this retrospective analysis were the influence of SII levels on progression-free (PFS) and overall survival (OS) results, respectively: defined as the intervals between the first day of the concurrent chemoradiotherapy and the first recorded date of disease progression or death/last visit for PFS and the date of death/last visit for OS. Medians and ranges were used for the quantitative variables, while categorical variables were described as frequencies and percentages, and were analyzed using Chi-square or Fisher's exact tests. The receiver operating characteristic (ROC) curve analysis was utilized to define the optimal cut-off values for SII that interact best with the PFS and OS outcomes. Pearson's *χ*^2^ test was carried out to perform comparisons between the demographic features of SII groups. Survival analyses and intergroup comparisons were performed using the Kaplan-Meier survival curves and two-sided Log-rank test analysis. Any 2-tailed *P* < 0.05 was considered significant. The multivariate Cox Proportional Hazard model was used to evaluate the relationship between different variables and survival outcomes by entering only the factors exhibiting significance in univariate analysis. Correlations between any two factors were tested with Pearson's exact test or Spearman's correlation analysis.

## 3. Results

### 3.1. Patient Demographics

We identified newly diagnosed and consecutively treated 192 GBM patients, but 25 were excluded from the analysis because of receiving hypofractionated short-course RT (*n* = 21) and self-refusal of concurrent TMZ treatment (*n* = 4); leaving 167 patients eligible for this analysis. Prechemoradiotherapy patient and disease characteristics for the entire study cohort were as summarized in Table 1. Median age was 57 (range: 26-80) with male gender (65.9%) and KPS 90-100 (%, 55.7%) dominancy. The median symptom duration was 2.1 months (range: 0.2-7.8 months). Subtotal excision (STR: 48.4%) followed by gross total excision (GTR: 35.3%) constituted the commonest surgical interventions. The overall corticosteroid and anticonvulsant usage rates at presentation were 67.1% and 34.1%, separately.

### 3.2. Optimal Cut-Off Value for SII

**Figure 1:**
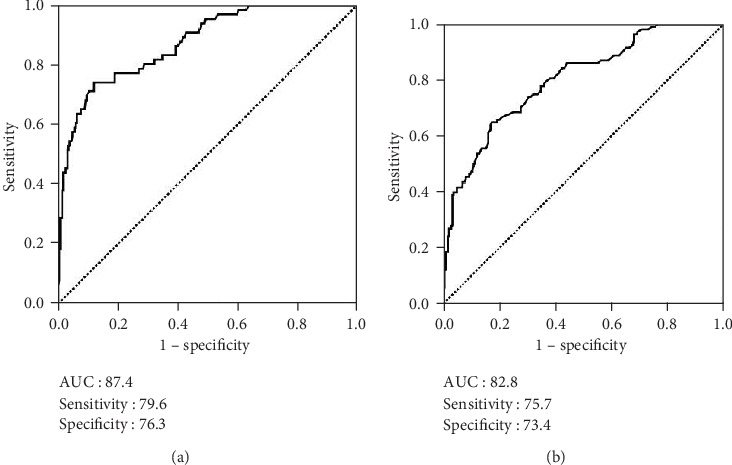
Receiver operating characteristic curve analyses outcomes. (a) Progression-free survival. (b) Overall survival.

We applied the ROC curve analysis as a more objective method for the search of optimal cut-offs for probable links between the SII and PFS and OS status, rather than the bias-prone mean/median values. The results of the ROC curve analysis exhibited the optimal cut-off values at 562 (area under the curve (AUC): 87.4%; sensitivity: 79.6%; and specificity: 76.3%) for PFS and at 569 (AUC: 82.8%; sensitivity: 75.7%; and specificity: 73.4%) for OS, respectively (Figure 1). Because the two cut-offs were very close, we used the rounded 565 value as the common cut-off for PFS and OS for stratification of patients into two groups for further analyses: low-SII (L-SII) group: SII ≤ 565 and high-SII (H-SII) group: SII > 565, respectively. Evaluation of the baseline demographics (Table 1) and salvage treatments (Table 2) per conveyance SII groups revealed no meaningful differences between the two cohorts, with only a tendency for higher corticosteroid use (75.9% versus 57.5%; *P* = 0.09) in the H-SII than the L-SII cohort.

### 3.3. Recurrence Patterns and Salvage Treatment

Seventeen (10.2%) patients were still alive at a median follow-up period of 13.8 months (range: 1.1-108.3 months), and 11 (6.6%) of them were free of disease progression. All 156 (93.4%) relapses were encountered intracranially (Table 2). Accounting for 92.9% (*n* = 145) of all relapse records, infield (*n* = 129; 82.6%) and marginal (*n* = 16; 10.3%) disease progressions constituted the commonest relapse forms. For the entire study cohort, the median and 5-year PFS and OS estimates were 9.0 months (95% confidence interval (CI): 7.2-10.8 months) and 3.8% for PFS and 14.4 months (95% CI: 11.9-16.9 months) and 9.8% for OS, separately (Table 2).

### 3.4. Association of SII with Survival Outcomes

**Figure 2:**
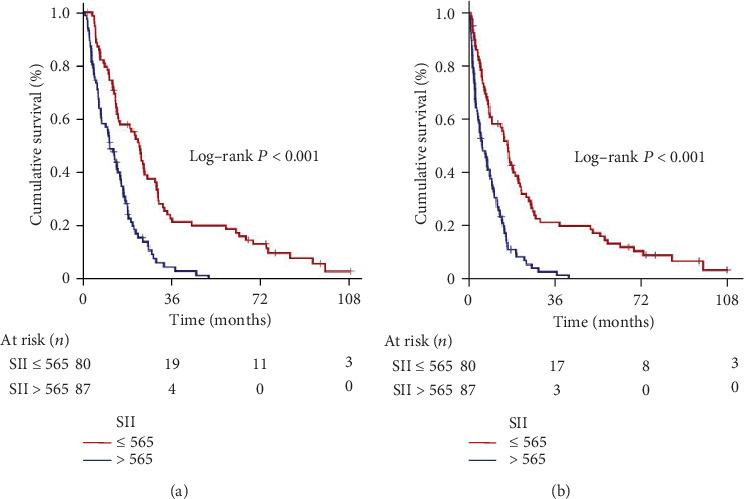
Survival outcomes per conveyance systemic immune-inflammation index groups. (a) Progression-free survival. (b) Overall survival (red: low systemic immune-inflammation index; dark blue: high systemic immune-inflammation index).

Granting the endpoints of the research, we compared the outcomes of patients allocated to the L-SII (*n* = 80) and H-SII (*n* = 87) groups in terms of PFS and OS. Results of comparative analyses paraded that the H-SII patients had significantly inferior median PFS (6.0 (95% CI: 3.1-8.9) versus. 16.6 (95% CI: 13.8-19.4)) and OS (11.1 (95% CI: 8.4-13.9) versus. 22.9 months (95% CI: 18.8-27.0)) than those patients with L-SII (Figure 2). Likewise, the 5-year PFS (0% versus 13.4%) and OS (0% versus 18.9%) estimates were also lower in the H-SII group (Table 2).

### 3.5. Outcomes of Univariate and Multivariate Analyses

Results of univariate analysis revealed the KPS 90-100 vs. 70-80 (*P* = 0.002 for PFS and *P* = 0.001 for OS), RTOG-RPA classes 3 vs. 4 vs. 5 (*P* < 0.001 for PFS and OS), gross total resection vs. subtotal resection/biopsy only (*P* = 0.006 for PFS and *P* = 0.009 for OS), and the L-SII vs. H-SII (*P* < 0.001 for PFS and OS) as the variables manifesting significant connection with the survival outcomes (Table 3). Among these factors, moreover, all four factors retained their independent association with the PFS and OS outcomes in multivariate analyses, as well: KPS (*P* = 0.008 for PFS and *P* = 0.005 for OS), RTOG-RPA class (*P* < 0.001 for PFS and OS), extent of neurosurgical intervention (*P* = 0.014 for PFS and *P* = 0.019 for OS), and SII grouping (*P* < 0.001 for PFS and OS), respectively (Table 3). Further analyses with Spearman's correlation tests among the factors exhibiting independent significance in multivariate analyses uncovered that the H-SII was meaningfully linked with a poorer performance status (KPS: 70-80; *r*_s_: -0.81for PFS and *r*_s_: -0.87 for OS) and higher RTOG-RPA classes (RTOG-RPA: IV-V; *r*_s_: -0.86 for PFS and *r*_s_: -0.94 for OS), but not the extent of resection (*r*_s_: -0.16 for PFS and *r*_s_: -0.22 for OS).

## 4. Discussion

The present study, to our best information, represents the first endeavor to particularly question the prognostic influence of SII on the survival outcomes of the newly diagnosed GBM patients treated with postneurosurgical RT plus concurrent and adjuvant TMZ. We demonstrated that the pretreatment H-SII was linked with significantly inferior median and 5-year PFS and OS rates than L-SII in this patients' group. Hence, adding to the well-recognized clinicopathologic factors, namely the KPS, RTOG-RPA, and extent of resection, our results proposed an adjunct robust and independent prognostic role for the novel inexpensive and clinically pertinent biomarker SII in the further prognostic lamination of GBM patients undergoing postoperative RT and TMZ. Additionally, present results revealed a meaningful correlation between an H-SII value and poorer KPS (70-80) and higher RTOG-RPA classes (IV-V), indicating a strong immune and inflammatory response in these particular patients groups.

As a novel finding for the modern GBM literature, present results convincingly showed that the pretreatment H-SII was strongly and independently associated with poorer median PFS (6.0 versus 16.6 months; *P* < 0.001) and OS (11.1 versus 22.9 months; *P* < 0.001) after the standard RT and TMZ combination. Albeit this is the first report in GBM patients to illustrate significant connections between the SII and survival results, they are harmonious with the outcomes of accessible SII researches and meta-analyses in other cancers [12–16]. In lack of GBM-specific research results, two recent notable studies carefully examined the correlation between the SII levels and glioma grades [18, 19], and proposing an increased systemic immune-inflammatory response by increasing glioma grade both reported that the SII was significantly higher in HGG than the LGG. Xu et al. reported that the mean SII was significantly higher in the HGG than the LGG (595.5 versus 488.1; *P* = 0.0016) group [18], while Liang et al. used the ROC curve analysis and found the 392.48 point as the most optimal cut-off SII value that discriminates HGG from LGG [19]. In our GBM cohort, the ROC curve analysis revealed the optimal cut-off at rounded 565 for PFS and OS endpoints, which is higher than Liang's 392.48 cut-off value. Albeit further studies are called for to define a more relevant GBM-specific SII cut-off, this distinction between two studies was not extraordinary, as we included exclusively the grade IV patients rather than the grade III and IV patients in the same pool: groups exhibiting remarkably different local and systemic immune and inflammatory responses.

The explicit mechanisms underlying the observation of a significant association between H-SII and poor GBM prognosis after curative therapy are currently not identified. Yet, the increased neutrophil and platelet counts and reversely decreased lymphocyte counts in the H-SII group rationally suggest that the prognostic distinction between the two SII groups might be the result of a depressed immunologic response against the heavily induced inflammatory status. Lymphocytes exert antigen-dependent and direct cytotoxic cell death and antiproliferative/antigrowth actions on tumor cells, rendering them the key components of the antitumor immunity [6, 20, 21]. In support, the higher magnitude of tumor-infiltrating lymphocytes has been shown to correlate with a better prognosis in GBM patients [22]. Conversely, neutrophils can promote tumor proliferation and growth by stimulating neoangiogenesis and induce a more malignant phenotype with increased levels of mesenchymal and other tumor progression markers: such as interleukin- (IL-) 3, IL-6, nitric oxide, and arginase [21]. Neutrophilia has also been proposed to be associated with higher tumor grade and, therefore, more aggressive tumor phenotypes [20]. In this respect, an experimental study showed that the increased recruitment of neutrophils was related to tumor grade, resistance to anti-VEGF therapy, and glioma progression with mesenchymal characteristics [23]. Furthermore, systemic inflammation may increase neutrophil counts and inversely decrease lymphocyte counts which may, regrettably, end up with a decreased cell-mediated cytotoxic immune response and resultant treatment failures [24]. Confirming the presence of the strongest inflammatory and the weakest immune response status in GBMs than other glioma grades, Zadora et al. showed that the NLR values were highest in GBMs compared to grade III (*P* < 0.01), grade II (*P* < 0.001), and grade I (*P* < 0.01) gliomas [25]. Platelets and platelet aggregates have been asserted to promote tumor progression [26]. It has been proven that platelet-derived TGF-*β* downregulates the cytokine NKG2D on the NK-cell surface to protect tumor cells from immune surveillance [27]. In coordination with TGF-*β*, platelets additionally activate the NF-*κ*B pathway via direct interactions with tumor cells and facilitate epithelial-mesenchymal transition: a major contributor to cellular migration, invasion, and metastasis [28]. Further evidence has also proposed that the platelet “cloak” that surrounds the tumor cells protects them from immune surveillance and renders them more prone to migration and metastasis [29]. Hence, the possible mechanisms underlying the H-SII in correlation with the poor prognoses of GBM patents might at least partially involve the consolidated effect of the increased neutrophil and platelet counts, accompanying decreased lymphocyte counts, as observed in our current study.

According to Clark [30], “a prognostic factor is a measure that relates to clinical results in the absence/presence of a standard therapy that patients are likely to receive.” Therefore, prognostic factors differ from predictive factors by their independence on particular therapies. To be built up clinically, a prognostic factor should fulfill the following key criteria: (1) reproducibly linked with a better or worse prognosis in clinics, (2) provided independent information in multivariate analyses among the other well-established factors, (3) reproduced objectively in multiple clinics or laboratories, and (4) scientifically proven prognostic incentive in prospective trials. Furthermore, to be useful in real-world practice, a prognostic factor ideally should also be affordable, easily achievable in routine tests or pathologic specimens, simply quantified or calculated, and relevant for all patients irrespective of their general condition. Considering these factors together, SII emerges to satisfy the criteria for being prognostic also for newly diagnosed GBM patients, as its contents are replicable and objectively measurable biochemical parameters that are readily available in routine biochemistry test panels with no excess cost, and applicable to any patients. Aligned with its persuasive power in the stratification of patients into two separate PFS and OS gatherings, such decent properties further render the SII a reasonable novel index for prognostic stratification of GBM patients planned to undergo RT plus TMZ.

The present study has some certain hindrances. First, it was a single-institutional retrospective cohort analysis in a comparatively small GBM cohort. Therefore, our discoveries ought to be interpreted with considerable caution until the compatible results of prospectively conceived corroborative large-scale studies become available. Second, nonattendance of the tumor-related variables, such as MGMT methylation status and isocitrate dehydrogenase-1 (IDH-1) and IDH-2, and local/systemic reactive proinflammatory cytokine/chemokine levels disallowed us to perform SII group-based analysis according to these biomarkers. In this regard, we rationally believe that future well-designed studies addressing these issues might provide valuable insights into the mechanistic relationship between these biomarkers and the SII in GBM patients. And last, although the SII was a dynamic biomarker that might have showed fluctuations during the treatment course with potential influences on the outcomes reported here, our analysis was restricted to the pretreatment SII. Studies focusing on the dynamics of SII during the whole treatment course may, therefore, serve valuable in terms of deciding the best-fit SII cut-off according to the time course.

## 5. Conclusions

The present first endeavor exploring the prognostic significance of SII on survival results of newly diagnosed GBM patients in the postneurosurgical concurrent chemoradiotherapy and adjuvant chemotherapy setting exhibited that the reproducibly measurable, cost-effective, and easily calculated H-SII levels were an adverse predictor of survival outcomes in this patients group. If verified with the results of future large-scale studies, such findings may demonstrate further valuable by contributing to the selection of the best-fit customized therapeutic strategies for GBM patients, particularly in the era of immunotherapy.

## Figures and Tables

**Table 1:** Baseline patient and disease characteristics.

Characteristic	All patients (*n* = 167)	L-SII (*n* = 80)	H-SII (*n* = 87)	*P* value
Median age, *y* (range)	57 (26-80)	59 (34-80)	58 (26-79)	0.83
Age group, *n* (%)				
<50 years	49 (29.3)	23 (28.7)	26 (29.9)	0.79
≥50 years	118 (70.7)	57 (71.3)	61 (70.1)	
Gender, *n* (%)				
Female	57 (34.1)	27 (33.7)	30 (34.5)	0.81
Male	110 (65.9)	53 (66.3)	57 (65.5)	
KPS, *n* (%)				
90-100	93 (55.7)	45 (56.3)	48 (55.1)	0.92
70-80	74 (44.3)	35 (43.7)	39 (44.9)	
RTOG-RPA class, *n* (%)				
III	65 (38.9)	30 (37.5)	35 (40.2)	0.67
IV	72 (43.1)	34 (42.5)	38 (43.7)	
V	30 (18.0)	16 (20.0)	14 (16.1)	
Symptom duration, *n* (%)				
<3 months	121 (72.5)	55 (68.8)	66 (75.8)	0.52
≥3 months	46 (27.5)	25 (31.2)	21 (24.1)	
Tumor location, *n* (%)				
Frontal	36 (21.6)	17 (21.3)	19 (21.9)	0.79
Parietal	30 (18.1)	14 (17.5)	16 (18.4)	
Temporal	34 (20.5)	16 (20.0)	18 (20.7)	
Occipital	17 (10.3)	10 (12.5)	7 (8.0)	
Midline	18 (10.2)	8 (10.0)	10 (11.5)	
Multilobar	32 (19.3)	15 (18.7)	17 (19.5)	
Extent of surgery, *n* (%)				
GTR	60 (35.9)	27 (33.7)	33 (40.2)	0.34
STR	76 (45.5)	37 (46.3)	39 (44.9)	
Biopsy	31 (18.6)	16 (20.0)	15 (14.9)	
Pre-RT T2-FLAIR volume				
<27.4 cc	82 (49.1)	38 (47.5)	44 (50.6)	0.73
≥27.4 cc	85 (50.9)	42 (52.5)	43 (49.4)	
Corticosteroid use, *n* (%)				
Yes	112 (67.1)	46 (57.5)	66 (75.9)	0.09
No	55 (22.9)	34 (42.5)	21 (24.1)	
Anticonvulsant use, *n* (%)				
Yes	57 (34.1)	31 (38.8)	26 (29.9)	0.28
No	110 (65.9)	49 (61.2)	61 (70.1)	

L-SII: low systemic immune-inflammation index; H-SII: high-SII; KPS: Karnofsky performance score; RTOG-RPA: Radiation Therapy Oncology Group recursive partitioning analysis; GTR: gross total resection; STR: subtotal resection; RT: radiotherapy; FLAIR: fluid attenuation inversion recovery.

**Table 2:** Treatment characteristics and clinical outcomes.

Characteristic	All patients (*n* = 167)	L-SII (*n* = 80)	H-SII (*n* = 87)	*P* value
RT technique, *n* (%)				
3D-CRT	93 (55.7)	45 (56.3)	48 (55.2)	0.79
SIB-IMRT	74 (44.3)	35 (43.7)	39 (44.8)	
RT dose, *n* (%)				
60 Gy	86 (51.5)	42 (52.5)	44 (50.6)	0.62
70 Gy	81 (48.5)	38 (47.5)	43 (49.4)	
Adjuvant TMZ cycles, *n* (%)				
1-5	48 (28.8)	23 (28.8)	25 (28.7)	0.96
6-12	119 (71.2)	57 (71.2)	62 (71.3)	
Brain failure, *n* (%)				
None	11 (6.6)	3 (3.8)	8 (9.2)	0.53
Infield	129 (77.3)	63 (78.8)	66 (75.9)	
Marginal	16 (9.6)	9 (11.2)	7 (8.0)	
Distant	6 (3.5)	2 (2.5)	4 (4.5)	
Infield and distant	3 (1.8)	2 (2.5)	1 (1.2)	
Marginal and distant	2 (1.2)	1 (1.2)	1 (1.2)	
Salvage treatment, *n* (%)				
None	76 (45.5)	36 (45.0)	40 (46.0)	0.54
Unknown	6 (3.6)	4 (5.0)	2 (2.4)	
SNS alone	17 (10.2)	8 (10.0)	9 (10.4)	
SRS/SRT	14 (8.4)	7 (8.7)	7 (7.9)	
SNS+SRS/SRT	8 (4.8)	3 (3.8)	5 (5.8)	
SNS+Ctx	15 (9.0)	8 (10.0)	7 (7.9)	
SNS+SRS+Ctx	7 (4.2)	3 (3.8)	4 (4.7)	
Ctx alone	24 (14.3)	11 (13.7)	13 (14.9)	
PFS				
Median, mo (95% CI)	9.0 (7.0-11.0)	16.6 (13.8-19.4)	6.0 (3.1-8.9)	<0.001
3 years (%)	11.9	21.4	2.8	
5 years (%)	6.6	13.4	0	
OS				
Median, mo (95% CI)	14.4 (11.9-16.9)	22.9 (18.8-27.0)	11.1 (8.4-13.9)	<0.001
3 years, %	14.0	23.0	4.7	
5 years, %	9.8	18.9	0	

L-SII: low systemic immune-inflammation index; H-SII: high-SII; RT: radiotherapy; TMZ: temozolomide; SNS: salvage neurosurgery; SRS: stereotactic radiosurgery; SRT: stereotactic radiotherapy; Ctx: chemotherapy; PFS: progression-free survival; OS: overall survival; CI: confidence interval.

**Table 3:** Results of uni- and multivariate analysis.

Variable	PFS			OS		
	Univariate *P* value	Multivariate *P* value	Hazard ratio	Univariate *P* value	Multivariate *P* value	Hazard ratio
Age (≤50 vs. >50 years)	0.17	—	—	0.14	—	—
Gender (male vs. female)	0.84	—	—	0.92	—	—
KPS (90-100 vs. 70-80)	0.002	0.008	1.48	0.001	0.005	1.57
RTOG-RPA group (III vs. IV vs. V)	<0.001	<0.001	1.98	<0.001	<0.001	2.14
Symptom duration (<3 vs. ≥3 months)	0.42	—	—	0.54	—	—
Extent of resection (GTR vs. STR/biopsy)	0.006	0.014	1.72	0.009	0.019	1.68
Pre-RT T2-FLAIR volume (<27 vs. ≥27 cc)	0.61	—	—	0.52	—	—
RT technique (3D-CRT vs. SIB-IMRT)	0.91	—	—	0.94	—	—
RT dose (60 vs. 70 Gy)	0.43	—	—	0.55	—	—
SII group (L-SII vs. H-SII)	<0.001	<0.001	2.07	<0.001	<0.001	2.77

PFS: progression-free survival; OS: overall survival; KPS: Karnofsky performance score; RTOG-RPA: Radiation Therapy Oncology Group recursive partitioning analysis; GTR: gross total resection; STR: subtotal resection; FLAIR: fluid attenuation inversion recovery; 3D-CRT: 3-dimensional conformal radiotherapy; SIB-IMRT: simultaneous integrated boost intensity-modulated radiotherapy; RT: radiotherapy; SII: systemic immune-inflammation index; L-SII: low-SII; H-SII: high-SII.

## Data Availability

Data is owned and saved by Baskent University Medical Faculty and, hence, cannot be shared without permission. Data are available from the Baskent University Radiation Oncology Institutional Data Access/Ethics Committee (contact via Baskent University Ethics Committee) for researchers meeting the criteria for access to confidential data: contact address, adanabaskent@baskent.edu.tr
